# Supplementary material for: Overlapping cell population expression profiling and regulatory inference in C. elegans
Source: BMC Genomics. 2016 Feb 29;17:159. doi: 10.1186/s12864-016-2482-z (PMC4772325; doi:10.1186/s12864-016-2482-z)
Supplement: Additional file 13: — Web supplement. (DOC 21 kb) [file 12864_2016_2482_MOESM13_ESM.zip › sortWeb/clusters/hier.300.clusters/5.html]

Cluster 5 

## Cluster 5

### Expression

| cnd-1 rep. 1 | cnd-1 rep. 2 | cnd-1 rep. 3 | pha-4 rep. 1 | pha-4 rep. 2 | pha-4 rep. 3 | ceh-27 | ceh-36 | ceh-6 | F21D5.9 | mir-57 | mls-2 | pal-1 | pros-1 | ttx-3 | unc-130 | hlh-16 | irx-1 | ceh-6 (+) hlh-16 (+) | ceh-6 (+) hlh-16 (-) | ceh-6 (-) hlh-16 (+) | cnd-1 singlets | pha-4 singlets | 0 | 60 | 120 | 150 | 180 | 240 | 330 | 390 | 420 | 480 | 540 | 570 | 600 | 630 | 660 | NAME | Functional description |
| --- | --- | --- | --- | --- | --- | --- | --- | --- | --- | --- | --- | --- | --- | --- | --- | --- | --- | --- | --- | --- | --- | --- | --- | --- | --- | --- | --- | --- | --- | --- | --- | --- | --- | --- | --- | --- | --- | --- | --- |
|  |  |  |  |  |  |  |  |  |  |  |  |  |  |  |  |  |  |  |  |  |  |  |  |  |  |  |  |  |  |  |  |  |  |  |  |  |  | F29A7.6 |  |
|  |  |  |  |  |  |  |  |  |  |  |  |  |  |  |  |  |  |  |  |  |  |  |  |  |  |  |  |  |  |  |  |  |  |  |  |  |  | *elc-1* | ELongin C |
|  |  |  |  |  |  |  |  |  |  |  |  |  |  |  |  |  |  |  |  |  |  |  |  |  |  |  |  |  |  |  |  |  |  |  |  |  |  | *rpoa-12* | RNA POlymerase I (A) subunit |
|  |  |  |  |  |  |  |  |  |  |  |  |  |  |  |  |  |  |  |  |  |  |  |  |  |  |  |  |  |  |  |  |  |  |  |  |  |  | *rnp-5* | RNP (RRM RNA binding domain) containing |
|  |  |  |  |  |  |  |  |  |  |  |  |  |  |  |  |  |  |  |  |  |  |  |  |  |  |  |  |  |  |  |  |  |  |  |  |  |  | Y67D2.7 |  |
|  |  |  |  |  |  |  |  |  |  |  |  |  |  |  |  |  |  |  |  |  |  |  |  |  |  |  |  |  |  |  |  |  |  |  |  |  |  | *rsp-6* | SR Protein (splicing factor) |
|  |  |  |  |  |  |  |  |  |  |  |  |  |  |  |  |  |  |  |  |  |  |  |  |  |  |  |  |  |  |  |  |  |  |  |  |  |  | *btb-6* | BTB (Broad/complex/Tramtrack/Bric a brac) domain protein |
|  |  |  |  |  |  |  |  |  |  |  |  |  |  |  |  |  |  |  |  |  |  |  |  |  |  |  |  |  |  |  |  |  |  |  |  |  |  | R09B3.2 |  |
|  |  |  |  |  |  |  |  |  |  |  |  |  |  |  |  |  |  |  |  |  |  |  |  |  |  |  |  |  |  |  |  |  |  |  |  |  |  | *rpa-2* | Replication Protein A homolog |
|  |  |  |  |  |  |  |  |  |  |  |  |  |  |  |  |  |  |  |  |  |  |  |  |  |  |  |  |  |  |  |  |  |  |  |  |  |  | Y69A2AR.21 |  |
|  |  |  |  |  |  |  |  |  |  |  |  |  |  |  |  |  |  |  |  |  |  |  |  |  |  |  |  |  |  |  |  |  |  |  |  |  |  | *tac-1* | TACC (transforming acid coiled coil) protein family |
|  |  |  |  |  |  |  |  |  |  |  |  |  |  |  |  |  |  |  |  |  |  |  |  |  |  |  |  |  |  |  |  |  |  |  |  |  |  | *ndx-6* | NuDiX family |
|  |  |  |  |  |  |  |  |  |  |  |  |  |  |  |  |  |  |  |  |  |  |  |  |  |  |  |  |  |  |  |  |  |  |  |  |  |  | *rsp-4* | SR Protein (splicing factor) |
|  |  |  |  |  |  |  |  |  |  |  |  |  |  |  |  |  |  |  |  |  |  |  |  |  |  |  |  |  |  |  |  |  |  |  |  |  |  | *spt-4* | SPT transcription factor family |
|  |  |  |  |  |  |  |  |  |  |  |  |  |  |  |  |  |  |  |  |  |  |  |  |  |  |  |  |  |  |  |  |  |  |  |  |  |  | T06A10.3 |  |
|  |  |  |  |  |  |  |  |  |  |  |  |  |  |  |  |  |  |  |  |  |  |  |  |  |  |  |  |  |  |  |  |  |  |  |  |  |  | *rpb-10* | RNA Polymerase II (B) subunit |
|  |  |  |  |  |  |  |  |  |  |  |  |  |  |  |  |  |  |  |  |  |  |  |  |  |  |  |  |  |  |  |  |  |  |  |  |  |  | *moag-4* | MOdifier of protein AGgregation |
|  |  |  |  |  |  |  |  |  |  |  |  |  |  |  |  |  |  |  |  |  |  |  |  |  |  |  |  |  |  |  |  |  |  |  |  |  |  | Y23H5A.2 |  |
|  |  |  |  |  |  |  |  |  |  |  |  |  |  |  |  |  |  |  |  |  |  |  |  |  |  |  |  |  |  |  |  |  |  |  |  |  |  | F54D7.6 |  |
|  |  |  |  |  |  |  |  |  |  |  |  |  |  |  |  |  |  |  |  |  |  |  |  |  |  |  |  |  |  |  |  |  |  |  |  |  |  | F37D6.3 |  |
|  |  |  |  |  |  |  |  |  |  |  |  |  |  |  |  |  |  |  |  |  |  |  |  |  |  |  |  |  |  |  |  |  |  |  |  |  |  | C18A3.9 |  |
|  |  |  |  |  |  |  |  |  |  |  |  |  |  |  |  |  |  |  |  |  |  |  |  |  |  |  |  |  |  |  |  |  |  |  |  |  |  | C33A11.2 |  |
|  |  |  |  |  |  |  |  |  |  |  |  |  |  |  |  |  |  |  |  |  |  |  |  |  |  |  |  |  |  |  |  |  |  |  |  |  |  | T25G12.11 |  |
|  |  |  |  |  |  |  |  |  |  |  |  |  |  |  |  |  |  |  |  |  |  |  |  |  |  |  |  |  |  |  |  |  |  |  |  |  |  | *tba-2* | TuBulin, Alpha |
|  |  |  |  |  |  |  |  |  |  |  |  |  |  |  |  |  |  |  |  |  |  |  |  |  |  |  |  |  |  |  |  |  |  |  |  |  |  | *his-32* | HIStone |
|  |  |  |  |  |  |  |  |  |  |  |  |  |  |  |  |  |  |  |  |  |  |  |  |  |  |  |  |  |  |  |  |  |  |  |  |  |  | C06E2.5 |  |
|  |  |  |  |  |  |  |  |  |  |  |  |  |  |  |  |  |  |  |  |  |  |  |  |  |  |  |  |  |  |  |  |  |  |  |  |  |  | *nhr-64* | Nuclear Hormone Receptor family |
|  |  |  |  |  |  |  |  |  |  |  |  |  |  |  |  |  |  |  |  |  |  |  |  |  |  |  |  |  |  |  |  |  |  |  |  |  |  | *str-225* | Seven TM Receptor |
|  |  |  |  |  |  |  |  |  |  |  |  |  |  |  |  |  |  |  |  |  |  |  |  |  |  |  |  |  |  |  |  |  |  |  |  |  |  | Y71F9B.13 |  |
|  |  |  |  |  |  |  |  |  |  |  |  |  |  |  |  |  |  |  |  |  |  |  |  |  |  |  |  |  |  |  |  |  |  |  |  |  |  | cTel55X.1 |  |
|  |  |  |  |  |  |  |  |  |  |  |  |  |  |  |  |  |  |  |  |  |  |  |  |  |  |  |  |  |  |  |  |  |  |  |  |  |  | *rib-1* | related to mammalian RIB-affecting EXT gene family |
|  |  |  |  |  |  |  |  |  |  |  |  |  |  |  |  |  |  |  |  |  |  |  |  |  |  |  |  |  |  |  |  |  |  |  |  |  |  | Y43D4A.10 |  |
|  |  |  |  |  |  |  |  |  |  |  |  |  |  |  |  |  |  |  |  |  |  |  |  |  |  |  |  |  |  |  |  |  |  |  |  |  |  | *exc-4* | EXCretory canal abnormal |
|  |  |  |  |  |  |  |  |  |  |  |  |  |  |  |  |  |  |  |  |  |  |  |  |  |  |  |  |  |  |  |  |  |  |  |  |  |  | ZK1320.9 |  |
|  |  |  |  |  |  |  |  |  |  |  |  |  |  |  |  |  |  |  |  |  |  |  |  |  |  |  |  |  |  |  |  |  |  |  |  |  |  | W09C3.4 |  |
|  |  |  |  |  |  |  |  |  |  |  |  |  |  |  |  |  |  |  |  |  |  |  |  |  |  |  |  |  |  |  |  |  |  |  |  |  |  | W02A11.1 |  |
|  |  |  |  |  |  |  |  |  |  |  |  |  |  |  |  |  |  |  |  |  |  |  |  |  |  |  |  |  |  |  |  |  |  |  |  |  |  | C11D2.4 |  |
|  |  |  |  |  |  |  |  |  |  |  |  |  |  |  |  |  |  |  |  |  |  |  |  |  |  |  |  |  |  |  |  |  |  |  |  |  |  | *exos-3* | EXOSome (multiexonuclease complex) component |
|  |  |  |  |  |  |  |  |  |  |  |  |  |  |  |  |  |  |  |  |  |  |  |  |  |  |  |  |  |  |  |  |  |  |  |  |  |  | Y71F9AL.12 |  |
|  |  |  |  |  |  |  |  |  |  |  |  |  |  |  |  |  |  |  |  |  |  |  |  |  |  |  |  |  |  |  |  |  |  |  |  |  |  | F10B5.2 |  |
|  |  |  |  |  |  |  |  |  |  |  |  |  |  |  |  |  |  |  |  |  |  |  |  |  |  |  |  |  |  |  |  |  |  |  |  |  |  | *fbxa-83* | F-box A protein |
|  |  |  |  |  |  |  |  |  |  |  |  |  |  |  |  |  |  |  |  |  |  |  |  |  |  |  |  |  |  |  |  |  |  |  |  |  |  | C37A2.6 |  |
|  |  |  |  |  |  |  |  |  |  |  |  |  |  |  |  |  |  |  |  |  |  |  |  |  |  |  |  |  |  |  |  |  |  |  |  |  |  | F56D1.2 |  |
|  |  |  |  |  |  |  |  |  |  |  |  |  |  |  |  |  |  |  |  |  |  |  |  |  |  |  |  |  |  |  |  |  |  |  |  |  |  | *ztf-13* | Zinc finger putative Transcription Factor family |
|  |  |  |  |  |  |  |  |  |  |  |  |  |  |  |  |  |  |  |  |  |  |  |  |  |  |  |  |  |  |  |  |  |  |  |  |  |  | F59G1.8 |  |
|  |  |  |  |  |  |  |  |  |  |  |  |  |  |  |  |  |  |  |  |  |  |  |  |  |  |  |  |  |  |  |  |  |  |  |  |  |  | *arp-6* | Actin-Related Proteins |
|  |  |  |  |  |  |  |  |  |  |  |  |  |  |  |  |  |  |  |  |  |  |  |  |  |  |  |  |  |  |  |  |  |  |  |  |  |  | *ubh-4* | UBiquitin C-terminal Hydrolase (family 1) |
|  |  |  |  |  |  |  |  |  |  |  |  |  |  |  |  |  |  |  |  |  |  |  |  |  |  |  |  |  |  |  |  |  |  |  |  |  |  | *dhhc-6* | DHHC-types zinc finger protein |
|  |  |  |  |  |  |  |  |  |  |  |  |  |  |  |  |  |  |  |  |  |  |  |  |  |  |  |  |  |  |  |  |  |  |  |  |  |  | *pad-2* | PAtterning Defective |
|  |  |  |  |  |  |  |  |  |  |  |  |  |  |  |  |  |  |  |  |  |  |  |  |  |  |  |  |  |  |  |  |  |  |  |  |  |  | Y104H12D.2 |  |
|  |  |  |  |  |  |  |  |  |  |  |  |  |  |  |  |  |  |  |  |  |  |  |  |  |  |  |  |  |  |  |  |  |  |  |  |  |  | Y104H12D.4 |  |
|  |  |  |  |  |  |  |  |  |  |  |  |  |  |  |  |  |  |  |  |  |  |  |  |  |  |  |  |  |  |  |  |  |  |  |  |  |  | *cdk-2* | Cyclin-Dependent Kinase family |
|  |  |  |  |  |  |  |  |  |  |  |  |  |  |  |  |  |  |  |  |  |  |  |  |  |  |  |  |  |  |  |  |  |  |  |  |  |  | B0238.11 |  |
|  |  |  |  |  |  |  |  |  |  |  |  |  |  |  |  |  |  |  |  |  |  |  |  |  |  |  |  |  |  |  |  |  |  |  |  |  |  | *dph-1* | DiPHthamide biosynthesis protein |
|  |  |  |  |  |  |  |  |  |  |  |  |  |  |  |  |  |  |  |  |  |  |  |  |  |  |  |  |  |  |  |  |  |  |  |  |  |  | C01G5.6 |  |
|  |  |  |  |  |  |  |  |  |  |  |  |  |  |  |  |  |  |  |  |  |  |  |  |  |  |  |  |  |  |  |  |  |  |  |  |  |  | *cdk-7* | Cyclin-Dependent Kinase family |
|  |  |  |  |  |  |  |  |  |  |  |  |  |  |  |  |  |  |  |  |  |  |  |  |  |  |  |  |  |  |  |  |  |  |  |  |  |  | Y82E9BR.17 |  |
|  |  |  |  |  |  |  |  |  |  |  |  |  |  |  |  |  |  |  |  |  |  |  |  |  |  |  |  |  |  |  |  |  |  |  |  |  |  | *tbb-1* | TuBulin, Beta |
|  |  |  |  |  |  |  |  |  |  |  |  |  |  |  |  |  |  |  |  |  |  |  |  |  |  |  |  |  |  |  |  |  |  |  |  |  |  | F13B12.1 |  |
|  |  |  |  |  |  |  |  |  |  |  |  |  |  |  |  |  |  |  |  |  |  |  |  |  |  |  |  |  |  |  |  |  |  |  |  |  |  | *cec-3* | C.Elegans Chromodomain protein |
|  |  |  |  |  |  |  |  |  |  |  |  |  |  |  |  |  |  |  |  |  |  |  |  |  |  |  |  |  |  |  |  |  |  |  |  |  |  | *dsb-2* | Double-Strand Break factor |
|  |  |  |  |  |  |  |  |  |  |  |  |  |  |  |  |  |  |  |  |  |  |  |  |  |  |  |  |  |  |  |  |  |  |  |  |  |  | *yif-1* | YIP1-Interacting Factor homolog |
|  |  |  |  |  |  |  |  |  |  |  |  |  |  |  |  |  |  |  |  |  |  |  |  |  |  |  |  |  |  |  |  |  |  |  |  |  |  | *pcyt-1* | Phosphocholine CYtidylylTransferase |
|  |  |  |  |  |  |  |  |  |  |  |  |  |  |  |  |  |  |  |  |  |  |  |  |  |  |  |  |  |  |  |  |  |  |  |  |  |  | C01G6.3 |  |
|  |  |  |  |  |  |  |  |  |  |  |  |  |  |  |  |  |  |  |  |  |  |  |  |  |  |  |  |  |  |  |  |  |  |  |  |  |  | *zhit-1* | Zinc finger, HIT-type |
|  |  |  |  |  |  |  |  |  |  |  |  |  |  |  |  |  |  |  |  |  |  |  |  |  |  |  |  |  |  |  |  |  |  |  |  |  |  | Y17G7B.3 |  |
|  |  |  |  |  |  |  |  |  |  |  |  |  |  |  |  |  |  |  |  |  |  |  |  |  |  |  |  |  |  |  |  |  |  |  |  |  |  | C24G6.8 |  |
|  |  |  |  |  |  |  |  |  |  |  |  |  |  |  |  |  |  |  |  |  |  |  |  |  |  |  |  |  |  |  |  |  |  |  |  |  |  | *spin-4* | SPINster (Drosophila lysosomal permease) homolog |
|  |  |  |  |  |  |  |  |  |  |  |  |  |  |  |  |  |  |  |  |  |  |  |  |  |  |  |  |  |  |  |  |  |  |  |  |  |  | Y111B2A.24 |  |
|  |  |  |  |  |  |  |  |  |  |  |  |  |  |  |  |  |  |  |  |  |  |  |  |  |  |  |  |  |  |  |  |  |  |  |  |  |  | *fbxc-36* | F-box C protein |
|  |  |  |  |  |  |  |  |  |  |  |  |  |  |  |  |  |  |  |  |  |  |  |  |  |  |  |  |  |  |  |  |  |  |  |  |  |  | K12H6.6 |  |
|  |  |  |  |  |  |  |  |  |  |  |  |  |  |  |  |  |  |  |  |  |  |  |  |  |  |  |  |  |  |  |  |  |  |  |  |  |  | *mff-2* | Mitochondrial Fission Factor |
|  |  |  |  |  |  |  |  |  |  |  |  |  |  |  |  |  |  |  |  |  |  |  |  |  |  |  |  |  |  |  |  |  |  |  |  |  |  | *gop-2* | Gro-1 OPeron gene |
|  |  |  |  |  |  |  |  |  |  |  |  |  |  |  |  |  |  |  |  |  |  |  |  |  |  |  |  |  |  |  |  |  |  |  |  |  |  | T07A9.8 |  |
|  |  |  |  |  |  |  |  |  |  |  |  |  |  |  |  |  |  |  |  |  |  |  |  |  |  |  |  |  |  |  |  |  |  |  |  |  |  | *tgt-2* | TRNA Guanine Transglycosylase |
|  |  |  |  |  |  |  |  |  |  |  |  |  |  |  |  |  |  |  |  |  |  |  |  |  |  |  |  |  |  |  |  |  |  |  |  |  |  | F57C2.5 |  |
|  |  |  |  |  |  |  |  |  |  |  |  |  |  |  |  |  |  |  |  |  |  |  |  |  |  |  |  |  |  |  |  |  |  |  |  |  |  | *npp-20* | Nuclear Pore complex Protein |
|  |  |  |  |  |  |  |  |  |  |  |  |  |  |  |  |  |  |  |  |  |  |  |  |  |  |  |  |  |  |  |  |  |  |  |  |  |  | F07E5.7 |  |
|  |  |  |  |  |  |  |  |  |  |  |  |  |  |  |  |  |  |  |  |  |  |  |  |  |  |  |  |  |  |  |  |  |  |  |  |  |  | F07E5.5 |  |
|  |  |  |  |  |  |  |  |  |  |  |  |  |  |  |  |  |  |  |  |  |  |  |  |  |  |  |  |  |  |  |  |  |  |  |  |  |  | *pus-1* | PseudoUridine Synthase family |
|  |  |  |  |  |  |  |  |  |  |  |  |  |  |  |  |  |  |  |  |  |  |  |  |  |  |  |  |  |  |  |  |  |  |  |  |  |  | F26E4.4 |  |
|  |  |  |  |  |  |  |  |  |  |  |  |  |  |  |  |  |  |  |  |  |  |  |  |  |  |  |  |  |  |  |  |  |  |  |  |  |  | C47E12.7 |  |
|  |  |  |  |  |  |  |  |  |  |  |  |  |  |  |  |  |  |  |  |  |  |  |  |  |  |  |  |  |  |  |  |  |  |  |  |  |  | C04F5.8 |  |
|  |  |  |  |  |  |  |  |  |  |  |  |  |  |  |  |  |  |  |  |  |  |  |  |  |  |  |  |  |  |  |  |  |  |  |  |  |  | *eif-3.I* | Eukaryotic Initiation Factor |
|  |  |  |  |  |  |  |  |  |  |  |  |  |  |  |  |  |  |  |  |  |  |  |  |  |  |  |  |  |  |  |  |  |  |  |  |  |  | ZK1127.5 |  |
|  |  |  |  |  |  |  |  |  |  |  |  |  |  |  |  |  |  |  |  |  |  |  |  |  |  |  |  |  |  |  |  |  |  |  |  |  |  | *nap-1* | Nucleosome Assembly Protein |
|  |  |  |  |  |  |  |  |  |  |  |  |  |  |  |  |  |  |  |  |  |  |  |  |  |  |  |  |  |  |  |  |  |  |  |  |  |  | F27D4.8 |  |
|  |  |  |  |  |  |  |  |  |  |  |  |  |  |  |  |  |  |  |  |  |  |  |  |  |  |  |  |  |  |  |  |  |  |  |  |  |  | T02C1.1 |  |
|  |  |  |  |  |  |  |  |  |  |  |  |  |  |  |  |  |  |  |  |  |  |  |  |  |  |  |  |  |  |  |  |  |  |  |  |  |  | *polg-1* | POLG (mitochondrial DNA POLymerase Gamma) |
|  |  |  |  |  |  |  |  |  |  |  |  |  |  |  |  |  |  |  |  |  |  |  |  |  |  |  |  |  |  |  |  |  |  |  |  |  |  | C56E6.2 |  |
|  |  |  |  |  |  |  |  |  |  |  |  |  |  |  |  |  |  |  |  |  |  |  |  |  |  |  |  |  |  |  |  |  |  |  |  |  |  | C41H7.3 |  |
|  |  |  |  |  |  |  |  |  |  |  |  |  |  |  |  |  |  |  |  |  |  |  |  |  |  |  |  |  |  |  |  |  |  |  |  |  |  | C41H7.6 |  |
|  |  |  |  |  |  |  |  |  |  |  |  |  |  |  |  |  |  |  |  |  |  |  |  |  |  |  |  |  |  |  |  |  |  |  |  |  |  | C41H7.4 |  |
|  |  |  |  |  |  |  |  |  |  |  |  |  |  |  |  |  |  |  |  |  |  |  |  |  |  |  |  |  |  |  |  |  |  |  |  |  |  | W09C5.1 |  |
|  |  |  |  |  |  |  |  |  |  |  |  |  |  |  |  |  |  |  |  |  |  |  |  |  |  |  |  |  |  |  |  |  |  |  |  |  |  | F32D1.5 |  |
|  |  |  |  |  |  |  |  |  |  |  |  |  |  |  |  |  |  |  |  |  |  |  |  |  |  |  |  |  |  |  |  |  |  |  |  |  |  | Y111B2A.9 |  |
|  |  |  |  |  |  |  |  |  |  |  |  |  |  |  |  |  |  |  |  |  |  |  |  |  |  |  |  |  |  |  |  |  |  |  |  |  |  | F59C12.4 |  |
|  |  |  |  |  |  |  |  |  |  |  |  |  |  |  |  |  |  |  |  |  |  |  |  |  |  |  |  |  |  |  |  |  |  |  |  |  |  | Y17G7B.12 |  |
|  |  |  |  |  |  |  |  |  |  |  |  |  |  |  |  |  |  |  |  |  |  |  |  |  |  |  |  |  |  |  |  |  |  |  |  |  |  | *dph-2* | DiPHthamide biosynthesis protein |
|  |  |  |  |  |  |  |  |  |  |  |  |  |  |  |  |  |  |  |  |  |  |  |  |  |  |  |  |  |  |  |  |  |  |  |  |  |  | T05H4.10 |  |
|  |  |  |  |  |  |  |  |  |  |  |  |  |  |  |  |  |  |  |  |  |  |  |  |  |  |  |  |  |  |  |  |  |  |  |  |  |  | C03H5.3 |  |
|  |  |  |  |  |  |  |  |  |  |  |  |  |  |  |  |  |  |  |  |  |  |  |  |  |  |  |  |  |  |  |  |  |  |  |  |  |  | *rpoa-49* | RNA POlymerase I (A) subunit |
|  |  |  |  |  |  |  |  |  |  |  |  |  |  |  |  |  |  |  |  |  |  |  |  |  |  |  |  |  |  |  |  |  |  |  |  |  |  | *trm-1* | TRNA Methylase |
|  |  |  |  |  |  |  |  |  |  |  |  |  |  |  |  |  |  |  |  |  |  |  |  |  |  |  |  |  |  |  |  |  |  |  |  |  |  | *cec-10* | C.Elegans Chromodomain protein |
|  |  |  |  |  |  |  |  |  |  |  |  |  |  |  |  |  |  |  |  |  |  |  |  |  |  |  |  |  |  |  |  |  |  |  |  |  |  | F13H10.3 |  |
|  |  |  |  |  |  |  |  |  |  |  |  |  |  |  |  |  |  |  |  |  |  |  |  |  |  |  |  |  |  |  |  |  |  |  |  |  |  | *ruvb-1* | RUVB (recombination protein) homolog |
|  |  |  |  |  |  |  |  |  |  |  |  |  |  |  |  |  |  |  |  |  |  |  |  |  |  |  |  |  |  |  |  |  |  |  |  |  |  | F25H2.12 |  |
|  |  |  |  |  |  |  |  |  |  |  |  |  |  |  |  |  |  |  |  |  |  |  |  |  |  |  |  |  |  |  |  |  |  |  |  |  |  | D1007.5 |  |
|  |  |  |  |  |  |  |  |  |  |  |  |  |  |  |  |  |  |  |  |  |  |  |  |  |  |  |  |  |  |  |  |  |  |  |  |  |  | *lipl-7* | LIPase Like |
|  |  |  |  |  |  |  |  |  |  |  |  |  |  |  |  |  |  |  |  |  |  |  |  |  |  |  |  |  |  |  |  |  |  |  |  |  |  | *rpl-11.1* | Ribosomal Protein, Large subunit |
|  |  |  |  |  |  |  |  |  |  |  |  |  |  |  |  |  |  |  |  |  |  |  |  |  |  |  |  |  |  |  |  |  |  |  |  |  |  | *exos-2* | EXOSome (multiexonuclease complex) component |
|  |  |  |  |  |  |  |  |  |  |  |  |  |  |  |  |  |  |  |  |  |  |  |  |  |  |  |  |  |  |  |  |  |  |  |  |  |  | ZK795.3 |  |
|  |  |  |  |  |  |  |  |  |  |  |  |  |  |  |  |  |  |  |  |  |  |  |  |  |  |  |  |  |  |  |  |  |  |  |  |  |  | T04A8.6 |  |
|  |  |  |  |  |  |  |  |  |  |  |  |  |  |  |  |  |  |  |  |  |  |  |  |  |  |  |  |  |  |  |  |  |  |  |  |  |  | Y53C12B.2 |  |
|  |  |  |  |  |  |  |  |  |  |  |  |  |  |  |  |  |  |  |  |  |  |  |  |  |  |  |  |  |  |  |  |  |  |  |  |  |  | *tag-267* | Temporarily Assigned Gene name |
|  |  |  |  |  |  |  |  |  |  |  |  |  |  |  |  |  |  |  |  |  |  |  |  |  |  |  |  |  |  |  |  |  |  |  |  |  |  | ZK1058.5 |  |
|  |  |  |  |  |  |  |  |  |  |  |  |  |  |  |  |  |  |  |  |  |  |  |  |  |  |  |  |  |  |  |  |  |  |  |  |  |  | *mars-1* | Methionyl Amino-acyl tRNA Synthetase |
|  |  |  |  |  |  |  |  |  |  |  |  |  |  |  |  |  |  |  |  |  |  |  |  |  |  |  |  |  |  |  |  |  |  |  |  |  |  | T07A9.14 |  |
|  |  |  |  |  |  |  |  |  |  |  |  |  |  |  |  |  |  |  |  |  |  |  |  |  |  |  |  |  |  |  |  |  |  |  |  |  |  | Y48G8AL.7 |  |
|  |  |  |  |  |  |  |  |  |  |  |  |  |  |  |  |  |  |  |  |  |  |  |  |  |  |  |  |  |  |  |  |  |  |  |  |  |  | *tag-296* | Temporarily Assigned Gene name |
|  |  |  |  |  |  |  |  |  |  |  |  |  |  |  |  |  |  |  |  |  |  |  |  |  |  |  |  |  |  |  |  |  |  |  |  |  |  | R09B3.3 |  |
|  |  |  |  |  |  |  |  |  |  |  |  |  |  |  |  |  |  |  |  |  |  |  |  |  |  |  |  |  |  |  |  |  |  |  |  |  |  | *iff-1* | Initiation Factor Five (eIF-5A) homolog |
|  |  |  |  |  |  |  |  |  |  |  |  |  |  |  |  |  |  |  |  |  |  |  |  |  |  |  |  |  |  |  |  |  |  |  |  |  |  | Y55F3AM.13 |  |
|  |  |  |  |  |  |  |  |  |  |  |  |  |  |  |  |  |  |  |  |  |  |  |  |  |  |  |  |  |  |  |  |  |  |  |  |  |  | *aqp-10* | AQuaPorin or aquaglyceroporin related |
|  |  |  |  |  |  |  |  |  |  |  |  |  |  |  |  |  |  |  |  |  |  |  |  |  |  |  |  |  |  |  |  |  |  |  |  |  |  | R07H5.8 |  |
|  |  |  |  |  |  |  |  |  |  |  |  |  |  |  |  |  |  |  |  |  |  |  |  |  |  |  |  |  |  |  |  |  |  |  |  |  |  | *mrpl-13* | Mitochondrial Ribosomal Protein, Large |
|  |  |  |  |  |  |  |  |  |  |  |  |  |  |  |  |  |  |  |  |  |  |  |  |  |  |  |  |  |  |  |  |  |  |  |  |  |  | *rab-18* | RAB family |
|  |  |  |  |  |  |  |  |  |  |  |  |  |  |  |  |  |  |  |  |  |  |  |  |  |  |  |  |  |  |  |  |  |  |  |  |  |  | *mrps-24* | Mitochondrial Ribosomal Protein, Small |
|  |  |  |  |  |  |  |  |  |  |  |  |  |  |  |  |  |  |  |  |  |  |  |  |  |  |  |  |  |  |  |  |  |  |  |  |  |  | F53F4.10 |  |
|  |  |  |  |  |  |  |  |  |  |  |  |  |  |  |  |  |  |  |  |  |  |  |  |  |  |  |  |  |  |  |  |  |  |  |  |  |  | *rab-8* | RAB family |
|  |  |  |  |  |  |  |  |  |  |  |  |  |  |  |  |  |  |  |  |  |  |  |  |  |  |  |  |  |  |  |  |  |  |  |  |  |  | T26E3.4 |  |
|  |  |  |  |  |  |  |  |  |  |  |  |  |  |  |  |  |  |  |  |  |  |  |  |  |  |  |  |  |  |  |  |  |  |  |  |  |  | C33C12.4 |  |
|  |  |  |  |  |  |  |  |  |  |  |  |  |  |  |  |  |  |  |  |  |  |  |  |  |  |  |  |  |  |  |  |  |  |  |  |  |  | *dcs-1* | DeCapping Scavenger enzyme homolog |
|  |  |  |  |  |  |  |  |  |  |  |  |  |  |  |  |  |  |  |  |  |  |  |  |  |  |  |  |  |  |  |  |  |  |  |  |  |  | *glrx-3* | GLutaRedoXin |
|  |  |  |  |  |  |  |  |  |  |  |  |  |  |  |  |  |  |  |  |  |  |  |  |  |  |  |  |  |  |  |  |  |  |  |  |  |  | *erd-2* | ERD (yeast endoplasmic reticulum retention defective) related |
|  |  |  |  |  |  |  |  |  |  |  |  |  |  |  |  |  |  |  |  |  |  |  |  |  |  |  |  |  |  |  |  |  |  |  |  |  |  | *iftb-1* | eIFTwoBeta (eIF2beta translation initiation factor) |
|  |  |  |  |  |  |  |  |  |  |  |  |  |  |  |  |  |  |  |  |  |  |  |  |  |  |  |  |  |  |  |  |  |  |  |  |  |  | *mrpl-41* | Mitochondrial Ribosomal Protein, Large |

### Phenotypes enriched

|  |  |  |  |
| --- | --- | --- | --- |
| **Group name** | **Number in cluster** | **Enrichment** | **FDR corrected p** |
| larval growth variant (RNAi) | 36 | 2.32 | 0.00162 |
| larval arrest (RNAi) | 35 | 2.26 | 0.00404 |
| developmental growth variant (RNAi) | 35 | 2.25 | 0.00409 |
| organism development variant (RNAi) | 63 | 1.66 | 0.00496 |
| lethal (RNAi) | 56 | 1.71 | 0.00938 |
| larval development variant (RNAi) | 35 | 2.09 | 0.01760 |
| development variant (RNAi) | 63 | 1.55 | 0.03780 |
| embryonic development variant (RNAi) | 50 | 1.69 | 0.04450 |
| postembryonic development variant (RNAi) | 35 | 1.98 | 0.04610 |

### Anatomy terms enriched

none found

### GO terms enriched

|  |  |  |
| --- | --- | --- |
| **GO term** | **Number of genes** | **FDR-corrected p-value** |
| embryo development ending in birth or egg hatching | 42 | 0.0038 |
| RNA processing | 9 | 0.0040 |
| ncRNA metabolic process | 7 | 0.0046 |
| intracellular | 36 | 0.0058 |
| cytoplasm | 26 | 0.0067 |
| nucleotidyltransferase activity | 5 | 0.0100 |
| anatomical structure development | 35 | 0.0170 |
| tRNA processing | 4 | 0.0190 |
| multicellular organismal process | 55 | 0.0240 |
| multicellular organismal development | 26 | 0.0390 |

### Expression clusters enriched

|  |  |  |  |
| --- | --- | --- | --- |
| **Group name** | **Number in cluster** | **Enrichment** | **FDR corrected p** |
| TGF- Dauer pathway adult transcriptional targets. Results obtained by comparing the microarray results of the dauer-constitutive mutants daf-7(e1372), daf-7(m62), and daf-1(m40) with dauer-defective mutants daf-3(mgDf90), daf-5(e1386), and daf-7(e1372);daf-3(mgDf90) double mutants at the permissive temperature, 20C, on the first day of adulthood. WBPaper00031040:TGF-beta\_adult\_downregulated | 89 | 2.43 | 1.17e-17 |
| Maternal-embryonic class (ME): genes that are in the intersection of the maternal and embryonic classes. | 68 | 2.94 | 1.05e-15 |
| Caenorhabditis elegans Genes with expression levels changed significantly after treatment of Bacillus thurigiensis DB27. | 92 | 2.20 | 1.21e-15 |
| Maternal class (M): genes that are called present in at least one of the three PC6 replicates. | 100 | 1.97 | 1.51e-14 |
| Embryonic class (E): genes that significantly increase in abundance at some point during embryogenesis. | 74 | 2.38 | 1.75e-12 |
| Caenorhabditis elegans Genes with expression levels changed significantly after treatment of Xenorhabdus nematophila. | 105 | 1.72 | 1.37e-11 |
| FBF-associated probe sets (FDR <2.25%) | 68 | 2.07 | 4.45e-08 |
| Genes significantly enriched (> 2x, FDR < 5%) in a particular cell-type versus a reference sample of all cells at the same stage. WBPaper00037950:bodywall-muscle\_larva\_enriched | 28 | 2.79 | 2.30e-04 |
| Genes expressed in N2. | 105 | 1.36 | 2.41e-04 |
| Germline-intrinsic transcripts. | 29 | 2.64 | 4.15e-04 |
| Genes with no change in hcf-1(-), no change in sir-2.1(O/E) and downregulated in daf-2(-). | 21 | 3.32 | 4.70e-04 |
| Genes up or down regulated by 10e-07M of progesterone. The normalized values used were G/R ratio > 2.6 for up-regulation and G/R ratio < 0.38 for down-regulation, which corresponds to 1.39 and -1.39 log(base2) G/R ratio, respectively. | 53 | 1.79 | 1.39e-03 |
| Genes expressed in embryonic motor neurons (identified by unc-4::GFP expressing cells). | 79 | 1.49 | 1.48e-03 |
| Genome-wide analysis of developmental and sex-regulated gene expression profile. cgc4489\_group\_4 | 15 | 3.93 | 2.10e-03 |
| Caenorhabditis elegans Genes with expression levels changed significantly after treatment of Serratia marcescens. | 54 | 1.75 | 2.14e-03 |
| C-lineage related expression profile. WBPaper00025032:cluster\_106 | 4 | 30.53 | 2.26e-03 |
| oogenesis-enriched | 24 | 2.60 | 4.12e-03 |
| Genes in the top 10% of expression level across the triplicate L3 samples. To generate the top10 and bottom10 gene sets, authors ranked all genes by mean expression array signal intensity across the three replicates, then took the top and bottom deciles (1,841 genes each) to represent genes with high and low expression. | 34 | 2.12 | 4.48e-03 |
| Differentially expressed genes during worm lifespan. Medoid 7 Fig.4. | 12 | 4.14 | 9.45e-03 |
| Expression Pattern Group H, enriched for genes involved in embryonic development. These patterns have in common that they all have genes of which the expression goes up after the juvenile stage. The expression of the genes in these patterns remains high or even goes up after reproduction. | 22 | 2.57 | 1.06e-02 |
| Embryonic (E) subclasses are based on the earliest significant increase(abbreviated pi for primary increase). [cgc5767]:expression\_class\_E\_pi(23\_min) | 20 | 2.54 | 2.63e-02 |
| Genes down regulated by mir-243(n4759). | 32 | 1.93 | 3.75e-02 |
| C-lineage related expression profile. WBPaper00025032:cluster\_2 | 9 | 4.52 | 3.96e-02 |

### Motifs enriched

|  |  |  |  |  |  |
| --- | --- | --- | --- | --- | --- |
| **Motif** | **Logo** | **Possible orthologs** | **Number of motifs in cluster** | **Enrichment** | **FDR corrected p** |
| Zfp161\_2858 |  | pzf-1 | 49 | 2.19 | 1.1e-05 |
| HLH29 |  | aha-1 lin-22 hlh-28 | 50 | 1.77 | 2.1e-03 |
| ARI3A\_do |  | cfi-1 | 105 | 1.28 | 3.5e-03 |
| pTH9958 |  | ztf-6 (0.64) | 56 | 1.64 | 3.8e-03 |
| pTH7876 |  | mel-28 (0.76) fkh-7 | 59 | 1.60 | 4.3e-03 |
| V$POU3F2\_01 |  | ceh-18 (-0.86) | 38 | 1.90 | 5.9e-03 |
| pTH9173 |  | efl-2 | 46 | 1.73 | 7.7e-03 |
| pTH7875 |  | mel-28 (0.76) | 72 | 1.44 | 8.7e-03 |
| pTH9026 |  | attf-1 | 35 | 1.93 | 8.9e-03 |
| pTH9262 |  | lin-54 | 91 | 1.32 | 9.8e-03 |
| pTH9335 |  | mel-28 (0.76) | 93 | 1.31 | 9.9e-03 |
| pTH9260 |  | mel-28 (0.76) | 70 | 1.45 | 1.1e-02 |
| MSX2\_f1 |  | ceh-1 | 58 | 1.55 | 1.1e-02 |
| MA0470.1 |  | F49E12.6 | 62 | 1.51 | 1.1e-02 |
| V$NKX61\_01 |  | cog-1 | 55 | 1.58 | 1.1e-02 |
| MA0041.1 |  | let-381 | 101 | 1.27 | 1.2e-02 |
| pTH10624 |  | Y61A9LA.9 Y116A8C.22 | 97 | 1.28 | 1.4e-02 |
| pTH9393 |  | ZC416.1 | 80 | 1.36 | 1.8e-02 |
| pTH9254 |  | mel-28 (0.76) | 50 | 1.59 | 2.0e-02 |
| ONECUT2\_1 |  | ceh-48 | 61 | 1.48 | 2.1e-02 |
| pTH9879 |  | hlh-26 hlh-30 C27D6.4 | 19 | 2.44 | 2.3e-02 |
| Mafk\_3106 |  | F45H11.6 | 79 | 1.35 | 2.3e-02 |
| MA0049.1 |  | hbl-1 | 104 | 1.23 | 2.7e-02 |
| pTH2280 |  | mnm-2 | 41 | 1.66 | 3.3e-02 |
| exd\_FlyReg\_FBgn0000611 |  | ceh-20 (0.57) | 100 | 1.24 | 3.4e-02 |
| Hey\_SANGER\_5\_FBgn0027788 |  | lin-22 | 12 | 3.13 | 3.5e-02 |
| pTH9222 |  | mel-28 (0.76) | 66 | 1.40 | 3.9e-02 |
| POU3F3\_3 |  | ceh-18 (-0.86) | 85 | 1.29 | 4.2e-02 |
| pTH5465 |  | hlh-30 | 23 | 2.07 | 4.2e-02 |
| pTH10696 |  | Y44A6D.3 | 38 | 1.67 | 4.5e-02 |
| SRP000712\_Sox2 |  | sox-4 (-0.63) | 94 | 1.25 | 4.6e-02 |
| pTH8556 |  | pax-2 | 14 | 2.69 | 4.7e-02 |
| MA0535.1 |  | daf-8 | 24 | 2.01 | 4.7e-02 |
| pTH4425 |  | cfi-1 | 75 | 1.34 | 4.8e-02 |

### Correlated (and anti-correlated) transcription factors

|  |  |
| --- | --- |
| **Transcription factor** | **Correlation** |
| sex-1 | 0.92 |
| dhhc-6 | 0.91 |
| Y53F4B.3 | 0.91 |
| duxl-1 | 0.89 |
| hmg-5 | 0.89 |
| Y82E9BR.17 | 0.89 |
| pop-1 | 0.88 |
| zip-7 | 0.87 |
| flh-3 | 0.85 |
| efl-3 | 0.84 |
| ces-2 | 0.84 |
| zim-1 | 0.84 |
| ekl-4 | 0.82 |
| dnj-11 | 0.82 |
| ztf-13 | 0.81 |
| hlh-2 | 0.81 |
| Y82E9BR.1 | 0.81 |
| flh-1 | 0.80 |
| F23A7.6 | 0.80 |
| F26F4.8 | 0.79 |
| ces-1 | 0.77 |
| F27D4.4 | 0.77 |
| C16A3.4 | 0.77 |
| ubxn-1 | 0.77 |
| R02D3.7 | 0.76 |
| ccch-1 | -0.65 |
| nhr-222 | -0.65 |
| nhr-197 | -0.65 |
| nhr-41 | -0.66 |
| ceh-54 | -0.67 |
| xbp-1 | -0.67 |
| mgl-2 | -0.67 |
| dmd-10 | -0.68 |
| gei-3 | -0.69 |
| nhr-40 | -0.69 |
| ceh-62 | -0.70 |
| nhr-198 | -0.71 |
| madf-4 | -0.73 |
| zip-1 | -0.74 |
| jun-1 | -0.74 |
| T18D3.7 | -0.74 |
| lfi-1 | -0.74 |
| zip-2 | -0.75 |
| tag-97 | -0.76 |
| ceh-88 | -0.76 |
| nhr-105 | -0.79 |
| zfh-2 | -0.83 |
| mdl-1 | -0.85 |
| ceh-18 | -0.86 |
| saeg-1 | -0.86 |

### ChIP peaks enriched

|  |  |  |  |  |
| --- | --- | --- | --- | --- |
| **Gene** | **Experiment** | **Number of upstream peaks** | **Enrichment** | **FDR corrected p** |
| efl-1 | EFL-1\_Young-adult | 87 | 2.71 | 7.1e-21 |
| efl-1 | EFL-1\_Fed-L1-stage-larvae | 74 | 3.22 | 1.3e-20 |
| lsy-2 | LSY-2\_Embryos | 65 | 3.62 | 5.9e-20 |
| dpl-1 | DPL-1\_Fed-L1-stage-larvae | 75 | 2.87 | 4.3e-18 |
| efl-1 | EFL-1\_Larvae-L1-stage | 78 | 2.68 | 2.6e-17 |
| lin-35 | LIN-35\_Fed-L1-stage-larvae | 68 | 3.01 | 8.1e-17 |
| dpl-1 | DPL-1\_Larvae-L4-stage | 83 | 2.37 | 1.2e-15 |
| W03F9.2 | W03F9.2\_L4-Young-Adult-stage-larvae | 91 | 2.14 | 4.2e-15 |
| hpl-2 | HPL-2\_Fed-L1-stage-larvae | 78 | 2.42 | 1.0e-14 |
| dpl-1 | DPL-1\_Young-adult | 64 | 2.87 | 1.7e-14 |
| lin-15 | LIN-15B\_Fed-L1-stage-larvae | 49 | 3.71 | 2.3e-14 |
| gei-11 | GEI-11\_Fed-L1-stage-larvae | 67 | 2.72 | 2.9e-14 |
| R02D3.7 | R02D3.7\_Larvae-L3-stage | 71 | 2.58 | 3.1e-14 |
| nfya-1 | NFYA-1\_Late-Embryos | 65 | 2.79 | 3.6e-14 |
| lsy-2 | LSY-2\_Larvae-L1-stage | 76 | 2.38 | 1.1e-13 |
| lsy-2 | LSY-2\_Fed-L1-stage-larvae | 66 | 2.68 | 1.2e-13 |
| F16B12.6 | F16B12.6\_Fed-L1-stage-larvae | 43 | 3.96 | 2.8e-13 |
| C34F6.9 | C34F6.9\_Larvae-L2-stage | 68 | 2.53 | 5.4e-13 |
| pes-1 | PES-1\_Larvae-L4-stage | 71 | 2.37 | 2.3e-12 |
| ceh-39 | CEH-39\_Embryos | 49 | 3.21 | 4.8e-12 |
| C16A3.4 | C16A3.4\_Fed-L1-stage-larvae | 53 | 2.92 | 1.3e-11 |
| C01B12.2 | C01B12.2\_Larvae-L2-stage | 79 | 2.00 | 2.4e-10 |
| F45C12.2 | F45C12.2\_Fed-L1-stage-larvae | 60 | 2.44 | 3.0e-10 |
| ham-1 | HAM-1\_Fed-L1-stage-larvae | 60 | 2.31 | 2.8e-09 |
| nhr-77 | NHR-77\_Fed-L1-stage-larvae | 58 | 2.36 | 2.8e-09 |
| fos-1 | FOS-1\_Fed-L1-stage-larvae | 57 | 2.39 | 2.9e-09 |
| eor-1 | EOR-1\_Larvae-L3-stage | 62 | 2.25 | 3.1e-09 |
| lin-35 | LIN-35\_Young-adult | 49 | 2.60 | 8.1e-09 |
| gei-11 | GEI-11\_Larvae-L3-stage | 58 | 2.29 | 9.9e-09 |
| lsy-2 | LSY-2\_Larvae-L2-stage | 37 | 3.13 | 2.4e-08 |
| lin-13 | LIN-13\_Larvae-L2-stage | 48 | 2.51 | 4.1e-08 |
| nhr-77 | NHR-77\_Larvae-L3-stage | 45 | 2.57 | 8.3e-08 |
| nfya-1 | NFYA-1\_Larvae-L3-stage | 52 | 2.32 | 9.4e-08 |
| nhr-237 | NHR-237\_Embryos | 32 | 3.17 | 3.3e-07 |
| nhr-77 | NHR-77\_Larvae-L4-stage | 71 | 1.82 | 5.5e-07 |
| F23B12.7 | F23B12.7\_Young-adult | 39 | 2.62 | 8.4e-07 |
| ces-1 | CES-1\_Embryos | 55 | 2.07 | 1.3e-06 |
| pha-4 | PHA-4\_Larvae-L2-stage | 59 | 1.97 | 1.6e-06 |
| nhr-23 | NHR-23\_Larvae-L3-stage | 50 | 2.17 | 1.7e-06 |
| ham-1 | HAM-1\_Larvae-L4-stage | 56 | 2.01 | 2.6e-06 |
| gei-11 | GEI-11\_Larvae-L2-stage | 43 | 2.29 | 5.6e-06 |
| R02D3.7 | R02D3.7\_Larvae-L4-stage | 32 | 2.75 | 7.5e-06 |
| ceh-38 | CEH-38\_Larvae-L3-stage | 48 | 2.12 | 8.1e-06 |
| lin-13 | LIN-13\_Larvae-L1-stage | 23 | 3.48 | 1.2e-05 |
| R02D3.7 | R02D3.7\_Larvae-L2-stage | 33 | 2.62 | 1.4e-05 |
| lin-15 | LIN-15B\_Larvae-L4-stage | 22 | 3.47 | 2.2e-05 |
| nhr-129 | NHR-129\_Larvae-L2-stage | 63 | 1.74 | 4.1e-05 |
| aly-2 | ALY-2\_Fed-L1-stage-larvae | 43 | 2.09 | 6.5e-05 |
| alr-1 | ALR-1\_Larvae-L2-stage | 45 | 2.01 | 8.8e-05 |
| nhr-25 | NHR-25\_Larvae-L2-stage | 43 | 2.04 | 1.1e-04 |
| ztf-7 | ZTF-7\_Larvae-L4-stage | 36 | 2.18 | 2.2e-04 |
| nhr-77 | NHR-77\_Larvae-L2-stage | 32 | 2.32 | 2.5e-04 |
| nhr-2 | NHR-2\_Embryos | 24 | 2.75 | 3.2e-04 |
| ama-1 | AMA-1\_Larvae-L3-stage | 27 | 2.49 | 4.5e-04 |
| gei-11 | GEI-11\_Young-adult | 28 | 2.42 | 4.8e-04 |
| sax-3 | SAX-3\_Larvae-L4-stage | 57 | 1.68 | 5.4e-04 |
| aly-2 | ALY-2\_Larvae-L3-stage | 30 | 2.31 | 5.7e-04 |
| zag-1 | ZAG-1\_Fed-L1-stage-larvae | 23 | 2.65 | 8.4e-04 |
| dve-1 | DVE-1\_Late-Embryos | 40 | 1.93 | 9.9e-04 |
| sem-4 | SEM-4\_Larvae-L2-stage | 46 | 1.77 | 1.5e-03 |
| lin-13 | LIN-13\_Larvae-L4-stage | 32 | 2.11 | 1.5e-03 |
| nhr-76 | NHR-76\_Larvae-L4-stage | 28 | 2.25 | 1.7e-03 |
| egl-5 | EGL-5\_Larvae-L3-stage | 35 | 1.99 | 1.9e-03 |
| nhr-6 | NHR-6\_Larvae-L4-stage | 33 | 2.00 | 3.0e-03 |
| zag-1 | ZAG-1\_Larvae-L2-stage | 32 | 2.03 | 3.0e-03 |
| jun-1 | JUN-1\_Larvae-L1-stage | 39 | 1.81 | 4.9e-03 |
| zag-1 | ZAG-1\_Larvae-L3-stage | 23 | 2.25 | 8.0e-03 |
| elt-3 | ELT-3\_Embryos | 33 | 1.83 | 1.3e-02 |
| pha-4 | PHA-4\_Larvae-L4-stage | 30 | 1.87 | 1.8e-02 |
| nhr-6 | NHR-6\_Larvae-L2-stage | 39 | 1.64 | 3.0e-02 |
| zag-1 | ZAG-1\_Larvae-L4-stage | 25 | 1.94 | 3.1e-02 |
| lsy-2 | LSY-2\_Larvae-L4-stage | 20 | 2.14 | 3.3e-02 |
| jun-1 | JUN-1\_Larvae-L4-stage | 28 | 1.80 | 4.4e-02 |
| hlh-30 | HLH-30\_Late-Embryos | 21 | 2.04 | 4.5e-02 |
| gei-11 | GEI-11\_Embryos | 18 | 2.19 | 4.5e-02 |
